# Of mosquitoes and men: mitigating Zika risk via Men’s family planning and male contraception

**DOI:** 10.1186/s40834-018-0069-6

**Published:** 2018-10-08

**Authors:** Brian T. Nguyen, Robyn Schickler

**Affiliations:** Section of Family Planning, Department of Obstetrics and Gynecology, Keck School of Medicine of the University of Southern California, Los Angeles, CA USA

**Keywords:** Male partner, Male contraception, men’s reproductive health, Zika, Family planning

## Abstract

**Background:**

Outbreaks of mosquito-borne viral illnesses commonly occur after storms. As storms are predicted to worsen in intensity and frequency, mosquito-borne viruses, including the Zika virus are expected to spread, and with devastating consequences. While the disease is self-limited, pregnant women who contract Zika can transmit the virus to their fetuses, causing neurodevelopmental abnormalities, including microcephaly. An overlooked vector of the Zika virus, however, is men whose semen can transmit the virus at the time of sexual intercourse. Current recommendations for preventing the sexual transmission of Zika are inadequate and need to emphasize male reproductive responsibility, via expanding services for men’s family planning and developing novel male contraceptives.

**Main body:**

To prevent the sexual transmission of Zika, the World Health Organization recommends that couples use condoms or abstain from sexual activity for at least 6 months when traveling in Zika-infected areas. Strict adherence to these recommendations is neither practical nor adequate to address Zika’s sexual transmission. As up to 13% of couples who use condoms experience unintended pregnancy, semen and consequent viral exposure is imminent. The use of contraception beyond just barrier methods is essential. However, the burden of contraception largely falls upon women and efforts to prevent vertical transmission are often aimed at educating women, when the outcome is equally undesirable among their male partners. These short-comings highlight the lack of attention to men’s options for family planning. Educating men about the full range of contraceptive options, correcting misconceptions about the efficacy of withdrawal and barrier contraceptive methods, increasing access to vasectomy, and developing novel male contraceptive options would allow shared responsibility for the prevention of unintended pregnancy and Zika-related morbidity.

**Conclusion:**

Gaps in recommendations to prevent the sexual transmission of Zika provide an opportunity to develop men’s family planning initiatives and the range of accessible contraceptives to men.

## Background

In 2017, the United States (US) confirmed its first cases of Zika infection in Texas and Florida, with 4 cases acquired through sexual transmission [1]. That same year, the US also fell victim to a record-breaking hurricane season [2]. As the National Oceanic and Atmospheric Administration projects that climate change will increase both the intensity and frequency of hurricanes along the Atlantic coast through the twenty-first century [3], and as populations continue to grow in urban, coastal areas [4], these circumstances will contribute to a public health “perfect storm.” Storms and their receding waters, particularly in regions that lack the resources and infrastructure for clean-up and vector control, leave stagnant pools that become breeding grounds for mosquitos that thrive and cause mosquito-borne viral infections [5], such as West Nile or Zika. After Hurricane Katrina (August 29, 2005), the incidence of West Nile Virus in storm-damaged regions more than doubled within 3 weeks [6]. While previous experience with disease outbreaks after hurricanes has spurred post-disaster education initiatives to prevent mosquito bites [7] and rapidly deployable vector control programs, such as aerial spraying [8], public funding for these programs is frequently strained, delays are not uncommon, and mosquitoes are becoming increasingly resistant to insecticides. These realities suggest that the spread of mosquito-borne Zika in the US will continue to be an area of concern as hurricanes continue to threaten the Atlantic coast in the coming years [9].

However, Zika presents a unique, additional challenge to public health because of an often overlooked, alternative vector for disease transmission—men, transmitting the virus to women via sexual contact with their semen. Given the risk of unintended pregnancy compounded by the risk of congenital anomaly among pregnancies infected by Zika, all routes of infection need to be addressed. A comprehensive, enduring Zika prevention plan should not only address the control of mosquitoes, but also examine the role of male sexual partners in the spread of Zika. Recommending that men practice abstinence and use condoms is not enough. Men should be actively educated about the risks of Zika and engaged in prevention efforts that emphasize the need for men to take on greater reproductive responsibility, as well as provide options for them to do so—comprehensive sex education, couples-inclusive contraceptive and abortion counseling, vasectomy, and the eventual development of novel male contraceptives.

## Main text

In 2016, the Center for Disease Control and Prevention (CDC) reported on the first case of asymptomatic male-to-female transmission of Zika in the US [10]. Sufficient evidence was accumulated in the same year linking severe neurodevelopmental anomalies, such as microcephaly, to the transmission of Zika from mother to fetus. Notably, a review of the CDC-sponsored US Zika Pregnancy Registry found that among 85 pregnancies with Zika exposure during the first trimester, 9 (11, 95%CI: 6–19%) exhibited neurodevelopmental defects [11]. These findings led the World Health Organization (WHO) to designate Zika as a Public Health Emergency of International Concern and issue a strategic response plan [12], specifically identifying the sexual partners of reproductive-age women as a target for education about the risk of and methods to prevent Zika transmission. As Zika can be detected in the semen for as many as 188 days, the WHO recommends that sexual partners traveling in and returning from Zika-infected areas (e.g., Brazil, Costa Rica, Cuba, Peru, Puerto Rico, etc.) should use condoms or abstain from sexual activity and avoid conceiving for no fewer than 6 months to avoid potentially transmitting Zika to sexual partners and/or pregnancies [13].

Adhering to recommendations to consistently use condoms or remain abstinent for 6 months, however, may be challenging for men, as well as for couples who do not or cannot use female methods of contraception. Further, the recommendation to start using condoms or adopt abstinence may not be realistic among couples in long-term or committed relationships. For adult couples who adopt condoms, the loss of sexual pleasure is reported by both men and women [14]. Further, a prospective study of couples using condoms over 4 weeks cited condom breakage or slippage among nearly 8% of couples [15], thereby raising doubts about the ability of couples to maintain perfect use of condoms. Consequently, among couples who use condoms to prevent pregnancy, 13% will typically experience an unintended pregnancy over the course of 1 year [16]. Regardless of whether this proportion is attributed to contraceptive failure or noncompliance, it highlights the incompleteness of recommendations for couples to rely solely on condoms to avoid pregnancy.

From a disease prevention standpoint, a survey of 520 Brazilian women noted that while nearly all women knew that Zika could be transmitted vertically, only 10.6% of pregnant women were using condoms to prevent sexual transmission [17]. While no study has examined rates of successful abstinence and condom use among couples following a possible Zika exposure, prospective research among sexually active, heterosexual, HIV-serodiscordant couples notes significant decreases in rates of abstinence and increases in rates of unprotected intercourse after 6 months of follow-up [18]. These findings suggest that “safe sex” messages may not be effective in preventing the spread of even a lethal sexually transmitted disease with known maternal-to-child transmission. As unsafe sex was more likely to occur in this study among couples where women were younger or less educated, disease transmission may be related to differences in power and reproductive agency or ineffective communication between women and their male partners. In some studies, women who report having contracted a sexually transmitted infection in the past continue to endorse difficulty with negotiating the use of condoms with their male sexual partners [19]. These concerns were supported in focus groups conducted among Brazilian women who claimed that men were either not aware of or concerned about the Zika risk, and that men would only use condoms if the women specifically demanded [20]. Given these findings, rather than relying solely on recommendations to use condoms or abstain, disease prevention may require a deeper understanding of men’s sexual and reproductive responsibility and relationship dynamics, as well as education and counseling initiatives to sensitize them to the risk that their behaviors, or lack of behaviors, pose to women.

Engaging men in the responsibility to abstain or use contraception is a gender equity issue, as women cannot bear the sole burden of preventing a pregnancy that could otherwise lead to a Zika-affected infant, particularly when the male partner in a relationship may very well be invested in the well-being of their child. The potential consequences of not sensitizing men to the need for contraception, with respect to the spread of Zika, are illustrated by the gender gap in women’s political empowerment in Brazil, which has widened over the past decade [21], leaving a political male majority, unaware of the value and need for access to both contraception and abortion. In Brazil, neither the levonorgestrel intrauterine system nor the subdermal contraceptive implant are subsidized by the government. A proposal to include these methods in the national health system was rejected by the Brazilian Ministry of Health on the grounds of high cost [22]. Additionally, the spread of Zika in Brazil and advisories about its linkage to microcephaly among infected infants led to a more than 100% increase in requests for abortions from Women on Web, an organization providing access to medical abortion in countries where abortion is not universally available or where abortion is illegal [23]. During this time, the Brazilian government was confiscating shipments of pills to be used for medical abortion and considered increasing the severity of sentences for women trying to obtain abortions to prevent the birth of infants with Zika-related anomalies [24], possibly contributing to nearly five thousand cases of microcephaly reported in Brazil from mid-2015 to early 2016 [25]. For men in political power in Brazil to restrict access to both contraception and abortion highlights their lack of both reproductive awareness and accountability. The case of Brazil provides a cautionary tale for legislators in other regions, such as Texas, where sexually-transmitted cases of Zika have been reported [26], and where funding for family planning services have been severely cut and numerous restrictions imposed on abortion [27]. As 80% of US congress members are men and as the Trump administration continues to threaten the provision of contraception by institutions such as Planned Parenthood [28], their awareness of the importance of family planning and its relevance to men is particularly important.

For men who recognize their role as vectors in the spread of Zika-related fetal anomalies, the shortcomings of barrier and female-controlled contraceptive methods may prompt them to consider their own options. For couples who no longer desire child-bearing, vasectomy may be considered. However, access to vasectomy in the US is limited by provider shortages in rural areas. Additionally, while US legislation via the Affordable Care Act mandates that health insurance providers cover essential preventative services such as female sterilization, vasectomy is explicitly excluded from the mandate, leaving logistic and surgical burdens on women [29].

The limits of men’s family planning services should prompt greater demand for new methods of male contraception. To date, contraceptive development has disproportionately focused on methods for women, with attempts to develop new male contraceptives largely abandoned by pharmaceutical companies due to concerns about side effects and the perceived lack of demand among men. In fact, Family Planning 2020, the global campaign committing numerous governments and foundations to ensuring women’s access to contraception and family planning, makes no mention of broadening contraceptive access for men [30], despite active conduct of human clinical trials on hormonal male contraceptive pills, injectables, implants, and topical gels [31], as well as research on multi-purpose contraceptives or spermicides with anti-viral activity [32]. The enduring oversight of men’s attention to family planning and their reproductive responsibility at both levels of industry and policy, contrasts enduring evidence from surveys of men. For example, an international survey of more than 9000 men in 2002 found willingness to use a novel method of male contraception among 50–83% of respondents [33]. Additionally, while the most recent international efficacy trial of a male hormonal contraceptive injection combining 200-mg of norethisterone enanthate combined with 1000-mg testosterone undecanoate was stopped early due to concerns related to the frequency of reported mood-related side effects, more than 80% of male participants endorsed their willingness to use this method of male contraception in the future [34]. In 2018, another international trial of a male hormonal contraceptive gel will begin [35], aimed at providing men with an easily applicable method with which they may be able to prevent an unintended pregnancy. Other non-hormonal methods of male contraception are also being studied, such as injections to reversibly occlude the vas deferens and drugs to interrupt the development or motility of sperm [36]. Their success will be dependent upon men’s recognition of the importance of their engagement in family planning. We hope that further efforts to improve men’s engagement in family planning and their contraceptive options will precede further threats to the women and children whom men aim to protect at all levels.

## Conclusions

While Zika is no longer a global emergency, the World Health Organization recognizes Zika as an enduring public health challenge. As neither an antiviral treatment nor a vaccine for Zika have emerged, prevention is the only viable strategy. The success of recommendations to prevent the spread of Zika both to women and their pregnancies however, is dependent upon the reproductive responsibility of and contraceptive access for both men and women. Recommendations for strict adherence to condoms or the maintenance of abstinence following Zika exposure and infection do not adequately recognize a reality for some women where abstinence may not be a choice, where the use of condoms cannot be negotiated with their male partner, or where their own contraceptives may be medically contraindicated. Effective strategies to mitigate the risk of Zika need to educate and engage men in their reproductive responsibility, educate them about the importance of contraception and abortion, as well as increase the range of contraceptives with which they can equally participate in prevention.

## Abbreviations

CDC: Centers for Disease Control and Prevention
WHO: World Health Organization
